# Modelo Preditivo de Mortalidade por Todas as Causas em Pacientes com Insuficiência Cardíaca Usando a Variabilidade da Frequência Cardíaca

**DOI:** 10.36660/abc.20220379

**Published:** 2023-12-07

**Authors:** Bruno Ferraz de Oliveira Gomes, Paulo Roberto Benchimol-Barbosa, Jurandir Nadal

**Affiliations:** 1 Hospital Barra D’Or Rio de Janeiro RJ Brasil Hospital Barra D’Or , Rio de Janeiro , RJ – Brasil; 2 Universidade Federal do Rio de Janeiro Rio de Janeiro RJ Brasil Universidade Federal do Rio de Janeiro , Rio de Janeiro , RJ – Brasil; 3 Universidade do Estado do Rio de Janeiro Hospital Universitário Pedro Ernesto Coordenação de Medicina Clínica Rio de Janeiro RJ Brasil Universidade do Estado do Rio de Janeiro , Hospital Universitário Pedro Ernesto – Coordenação de Medicina Clínica , Rio de Janeiro , RJ – Brasil; 4 Universidade Federal do Rio de Janeiro Instituto Alberto Luiz Coimbra de Pós-graduação e Pesquisa de Engenharia Programa de Engenharia Biomédica Rio de Janeiro RJ Brasil Universidade Federal do Rio de Janeiro Instituto Alberto Luiz Coimbra de Pós-graduação e Pesquisa de Engenharia – Programa de Engenharia Biomédica , Rio de Janeiro , RJ – Brasil

**Keywords:** Frequência Cardíaca, Insuficiência Cardíaca, Mortalidade

## Abstract

**Fundamento:**

Dados de curto e de longo prazo da variabilidade da frequência cardíaca (VFC) poderiam identificar preditores de mortalidade por todas as causas em pacientes com insuficiência cardíaca (IC).

**Objetivos:**

Construir um modelo preditivo de mortalidade por todas as causas em pacientes com IC usando a VFC.

**Métodos:**

Estudo retrospectivo incluindo pacientes com suspeita ou diagnóstico confirmado de IC internados por IC descompensada ou síncope e que realizaram exame de Holter 24 horas. Na análise do tônus simpático aumentado, nós avaliamos a VFC mais baixa em períodos de 10 minutos não sobrepostos em um registro contínuo de sinal eletrocardiográfico por 24 horas (VFC de curta duração). As variáveis com p<0,01 foram incluídas no modelo de regressão multivariada de Cox para determinar a ocorrência da mortalidade por todas as causas. As variáveis com significância estatística na regressão de Cox foram escolhidas para construir o modelo preditivo. Um p<0,05 foi considerado estatisticamente significativo.

**Resultados:**

Um total de 116 pacientes foram incluídos, com idade média de 71,9±16,3 anos, 45,7% eram do sexo masculino. O tempo médio de acompanhamento foi de 2,83 ± 1,27 anos. Trinta e nove (33,6%) óbitos ocorreram. Na comparação de sobreviventes e não sobreviventes, as variáveis que mostraram significância estatística foram menor SDNN, menor rMSSD, idade e fração de ejeção ventricular esquerda (FEVE). Na regressão Cox, os preditores independentes de mortalidade por todas as causas foram: idade > 69 anos (HR 3,95, IC95% 1,64-9,52); FEVE≤57% (HR 4,70, IC95% 2,38-9,28) e menor rMSSD ≤12ms (HR 5,54; IC 95% 2,04-15,08). Um valor inteiro foi atribuído para cada variável. Este escore < 3 apresentou uma área sob a curva de 0,802 (IC95% 0,72-0,87).

**Conclusão:**

Em pacientes com IC internados por IC descompensada ou síncope, preditores de longo prazo de mortalidade por todas as causas foram idade, FEVE, e rMSSD em 10 minutos. Esses achados indicam que mesmo breves momentos de tônus simpático elevado podem ter impacto na sobrevida, principalmente em idosos e pacientes com IC e fração de ejeção reduzida.

## Graphical abstract

**Figura Central:**
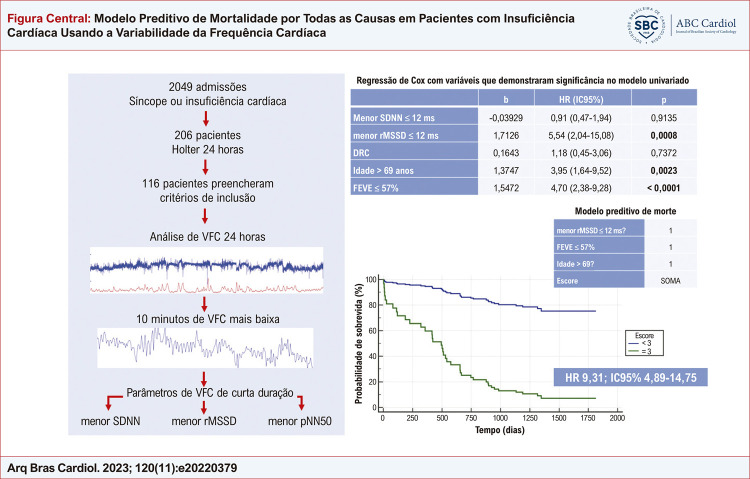
: Modelo Preditivo de Mortalidade por Todas as Causas em Pacientes com Insuficiência Cardíaca Usando a Variabilidade da Frequência Cardíaca

## Introdução

O sistema nervoso autônomo (SNA) é responsável por manter a homeostase do corpo. Muitas doenças, incluindo insuficiência cardíaca (IC), podem causar distúrbios no SNA, comprometendo a homeostase, e gerando mudanças na fisiologia cardiovascular. ^1^

Estudos experimentais mostraram que uma melhora na influência parassimpática sobre o coração tem um efeito antiarrítmico e antifibrilatório, ^2 , 3^ enquanto a atividade simpática é geralmente pró-arrítmica. ^3^ Assim, a presença de uma dominância simpática associada a outro processo pró-arrítmico (infarto do miocárdio ou IC), pode aumentar o risco de morte súbita.

Apesar do grande número de testes capazes de avaliar a função autonômica do indivíduo, há poucos dados mostrando quais testes ou combinações seriam os mais apropriados em diferentes situações clínicas. ^4^

A variabilidade da frequência cardíaca (VFC) é definida como a variação no intervalo RR em ritmo sinusal, e reflete o status autonômico na regulação da frequência cardíaca (FC). Estudos prévios associaram uma baixa VFC com mortalidade em pacientes após infarto do miocárdio, na IC, na neuropatia diabética, e após transplante cardíaco. ^5^

Quando analisadas usando o Holter 24 horas, as variáveis da VFC expressam o comportamento da FC por um longo período. Medidas de 24 horas em longo prazo não detectam situações breves de aumento no tônus simpático. Assim, o estudo das variáveis de curto prazo durante o momento de maior tônus simpático (menor VFC) pode aumentar a capacidade preditiva de morte nesses pacientes. Este estudo tem como objetivo identificar os preditores de mortalidade em pacientes com IC usando dados clínicos e dados de VFC de curto e de longo prazo obtidos por monitoramento por Holter 24 horas a fim de criar um modelo preditivo de mortalidade por todas as causas nesses pacientes.

## Delineamento do estudo

### População

Estudo retrospectivo do tipo coorte com pacientes admitidos por IC descompensada ou síncope em dois hospitais privados com suspeita ou diagnóstico confirmado de IC. A seleção dos pacientes com síncope foi necessária, uma vez que esses pacientes são rotineiramente submetidos ao monitoramento por Holter 24h. Apesar de ter um mecanismo fisiopatológico diferente, a síncope pode ser um marcador de eventos arrítmicos, e estar envolvido em um risco aumentado de morte. ^6^ O período do estudo foi de janeiro de 2014 a dezembro de 2016. Foram identificados pacientes que se submeteram ao Holter durante a internação ou após a alta hospitalar (até 30 dias) em um banco de dados com 4179 exames. Pacientes com IC terminal, evidência de outra doença cujo prognóstico apresenta uma expectativa de sobrevida inferior a um ano, pacientes com síndrome coronária aguda em menos de dois meses, registros de Holter com duração menor que 18 horas, ritmo não sinusal, marca-passo, e total de batimentos prematuros (atrial + ventricular) ≥ 5000 foram excluídos.

Dados clínicos, laboratoriais e ecocardiográficos dos pacientes considerados elegíveis foram avaliados a partir dos prontuários médicos, e relatórios de admissão e de alta. Após essa análise, o paciente foi classificado como um paciente sem IC (sem sintomas compatíveis com IC ou ecocardiograma sem disfunção sistólica e diastólica ou níveis de peptídeo natriurético tipo-B (BNP) abaixo da referência do laboratório ou um paciente com IC.

### Análise dos sinais

Os sinais foram adquiridos pelo aparelho de Holter de três canais, modelo DR200/HE (NorthEast Monitoring, MA, EUA). A largura de banda dos registros varia de 0,05 a 70Hz com 12 bits de resolução, e taxa de amostragem de 180 amostras por segundo. O processamento do sinal foi realizado usando um programa baseado no aplicativo Matlab® (The MathWorks, MA, EUA). Os indicadores da VFC foram registrados por dois observadores cegos para os desfechos, em duas situações: parâmetros tradicionais de longo e de curto prazo. Nos parâmetros de longo prazo, as seguintes variáveis foram avaliadas: SDNN 24h (desvio padrão de todos os intervalos RR normais gravados em 24 horas) SDANN 24h (desvio padrão das médias dos intervalos RR normais, a cada 5 minutos, em 24 horas); rMSSD 24h (raiz quadrada da média do quadrado das diferenças entre intervalos RR normais adjacentes em 24 horas), FC média, batimentos ventriculares prematuros (BVP) e batimentos supraventriculares prematuros.

Os registros do Holter 24 horas foram segmentados em janelas de 10 minutos sem sobreposição, e a janela com a VFC mais baixa foi identificada para avaliar os parâmetros de curta duração (momento de maior tônus simpático). Cada janela de 10 minutos foi examinada individualmente e incluídas na análise se as extrassístoles, os artefatos, e as batidas normais inadequadas, em conjunto, correspondesse a menos de 5% da duração da janela. As variáveis de curta duração geradas foram: menor SDNN (menor SDNN registrado em uma janela de 10 minutos durante o sinal de 24 horas); menor rMSSD (menor rMSSD registrado em uma janela de 10 minutos durante o sinal de 24 horas), e menor pNN50 (menor pNN50 registrado em uma janela de 10 minutos durante o sinal de 24 horas).

### Variáveis clínicas

Idade, sexo, causa da IC, comorbidades, e uso prévio de medicamentos foram avaliados. Dados laboratoriais e registros ecocardiográficos também foram avaliados; o primeiro ecocardiograma na admissão foi considerado na análise.

### Desfechos

O primeiro desfecho do estudo foi mortalidade por todas as causas. O período mínimo de acompanhamento foi de 12 meses.

### Aspectos éticos

O protocolo do estudo foi registrado na Plataforma Brasil com CAAE 63827617.5.0000.5249 após aprovação pelos comitês de ética local do Hospital Copa D’Or em 27/04/2017. Como esse era um estudo observacional retrospectivo, o comitê de ética isentou-o do termo de consentimento informado.

### Análise estatística

Os resultados foram expressos em média ± desvio padrão (distribuição normal) ou mediana e intervalo interquartil (distribuição não normal) para as variáveis contínuas e número de ocorrências (com porcentagem) para as variáveis categóricas. Características clínicas, laboratoriais, ecocardiográficas e variáveis da VFC dos pacientes vivos e os óbitos foram avaliados. O teste do qui-quadrado para as variáveis categóricas, e o teste t de Student não pareado para as variáveis contínuas foram usados. Quando a distribuição da amostra não era normal de acordo com o teste de Shapiro-Wilk, o teste de Mann-Whitney foi usado. Todas as variáveis com p<0,05 foram avaliados usando um modelo de regressão de Cox. As variáveis com p<0,01 no modelo univariado foram incluídas em um modelo de regressão multivariada de Cox para determinar a ocorrência de morte por todas as causas. As variáveis da VFC com p<0,05 no modelo de Cox para o desfecho mortalidade também foram avaliadas pela curva ROC para determinar o ponto de corte ótimo, a fim de criar um escore de modelo preditivo. Para a adição do ponto de corte, cada variável recebeu o valor inteiro correspondente a seu coeficiente beta obtido na regressão de Cox. As curvas de sobrevida foram apresentadas nos subgrupos de acordo com o modelo preditivo.

## Resultados

### Fluxo de inclusão dos pacientes no estudo

**Figura 1:**
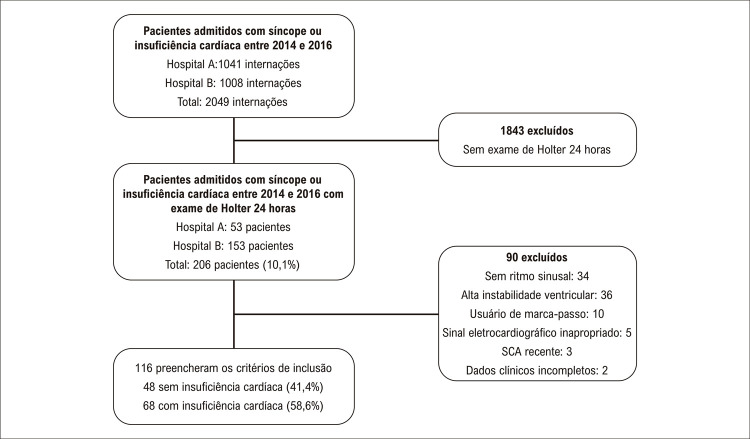
– Fluxograma da inclusão dos pacientes no estudo.

O fluxo de inclusão foi resumido na Figura 1 . Houve 2049 internações em ambos os hospitais; desses pacientes, 206 (10,1%) realizaram Holter. Noventa pacientes foram excluídos da amostra, totalizando 116 pacientes para a avaliação clínica. Após essa avaliação, 48 (41,4%) pacientes com função cardíaca compensada durante a internação e 68 (58,6%) com IC foram identificados. Sessenta e dois (53,4%) foram admitidos por síncope.

### Características da população

As características da população estão resumidas na Tabela 1 . Houve uma predominância de idosos e de mulheres. A comorbidade mais prevalente foi hipertensão arterial, seguida de diabetes e doença cardíaca isquêmica.

**Tabela 1:** – Características clínicas

	Todos (n=116)	Sem IC (n=48)	IC (n=68)	Valor p*
Idade (anos)	71,9 ± 16,3	68,3 ± 21,3	74,5 ± 11,0	0,042
Sexo masculino	53 (45,7%)	15 (31,3%)	38 (55,9%)	0,007
Hipertensão arterial	90 (77,6%)	31 (64,6%)	59 (89,4)	0,001
Diabetes	39 (33,6%)	9 (18,8%)	30 (44,1%)	0,004
Doença cardíaca isquêmica	21 (18,1%)	0 (0,0%)	21 (31,8%)	<0,001
Insuficiência renal crônica	13 (11,2%)	1 (7,7%)	12 (17,6%)	0,007
Acidente vascular cerebral	10 (8,6%)	2 (4,2%)	8 (11,8%)	0,135
FEVE (%)	56,3±17,2	68,5±6,8	47,7±17,2	<0,001

IC: insuficiência cardíaca; FEVE: fração de ejeção do ventrículo esquerdo; *comparação entre IC e sem IC.

Em relação à função ventricular, a maioria dos pacientes apresentou função sistólica ventricular esquerda preservada. O uso de betabloqueadores só foi identificado em um terço da população. Poucos pacientes manifestaram taquicardia ventricular não sustentada no Holter.

A duração média do Holter foi 23,23 ± 1,48 horas, e o seguimento médio foi de 2,83 ± 1,27 anos. A maioria dos exames de Holter foi realizado no hospital e metade na unidade de terapia intensiva. Ocorreram 39 óbitos.

As características também foram avaliadas conforme a classificação final dos pacientes (com ou sem IC). Não houve diferença de idade entre os grupos. Houve uma maior prevalência de homens em portadores de IC, assim como foi observada maior prevalência de hipertensão arterial, diabetes, doença isquêmica do coração e insuficiência renal crônica nessa população.

### Variabilidade da frequência cardíaca entre os grupos

Os pacientes com IC apresentaram parâmetros de VFC reduzidos em comparação aos pacientes sem IC. Não houve diferença na FC média entre esses dois grupos. O número de BVP foi maior nos pacientes com IC ( Tabela 2 ).

**Tabela 2:** – Variabilidade da frequência cardíaca entre os grupos

	Sem IC (n=48)	IC (n=68)	Valor p
SDNN 24h (ms)	88 (71-128)*	83,5 (59-109)*	0,061
SDANN 24h (ms)	79,5 (64-112)*	71,5 (49,5-91)*	0,037
rMSSD 24h (ms)	27 (15,25-43,25)*	24,5 (18-35,5)*	0,723
Menor SDNN (ms)	16,5 (11,5-24,5)*	13,0 (8,5-16)*	0,002
Menor rMSSD (ms)	10 (8-19,5)*	9 (7-13,5)*	0,070
menor pNN50 (%)	0,01 (0,001-1,17)*	0,01 (0,001-0,17)*	0,040
24h FC (bpm) média	72 (64-76,5)*	68 (62-76)*	0,159
BVP	8 (0-74,8)*	152 (7-553)*	<0,001
BSP	48 (3,75-201)*	70,5 (12-318)*	0,347

SDNN 24h: desvio padrão de todos os intervalos RR normais gravados em 24 horas; SDANN 24h: desvio padrão das médias dos intervalos RR normais, a cada 5 minutos, em 24 horas; rMSSD 24h: raiz quadrada da média do quadrado das diferenças entre intervalos RR normais adjacentes em 24 horas, IC: insuficiência cardíaca; BVP: batimentos ventriculares prematuros; BSP: batimentos supraventriculares prematuros; FC: frequência cardíaca. * mediana e intervalo interquartil.

### Variáveis com impacto sobre mortalidade

Os resultados relacionados à mortalidade por todas as causas estão descritos na Tabela 3 . A idade média foi mais alta no grupo de pacientes que foram a óbito, bem como a prevalência de insuficiência renal crônica, e fração de ejeção do ventrículo esquerdo (FEVE) reduzida, e esses fatores foram estatisticamente significativos na regressão de Cox univariada. Quanto aos parâmetros da VFC, aqueles que mostraram significância estatística na regressão univariada de Cox foram menor SDNN e menor rMSSD (p<0,05 para ambos).

**Tabela 3:** – Análise univariada para o desfecho morte por todas as causas

	Sobreviventes (n = 77)	Óbitos (n = 39)	p	Regressão de Cox Exp(b); (p)
Idade média (anos)	68,6 ± 18,0	78,4 ± 9,6	0,002	1,04 (p = 0,003)
Sexo masculino	32 (41,6%)	21 (53,8%)	0,145	
Hipertensão	58 (75,3%)	32 (86,5%)	0,130	
Diabetes	24 (31,2%)	15 (38,5%)	0,280	
IM prévio	14 (18,2%)	7 (18,9%)	0,557	
DRC	3 (3,9%)	10 (25,6%)	0,001	4,5 (p<0,001)
AVC prévio	4 (5,2%)	6 (15,4%)	0,070	
Uso de betabloqueador	24 (31,2%)	14 (35,9%)	0,378	
FEVE (%)	59,9 ± 15,9	49,3 ± 17,7	0,002	0,98 (p = 0,003)
Menor SDNN (ms)	18,7 ± 11,4	12,3 ± 5,2	0,001	0,90 (p = 0,001)
Menor rMSSD (ms)	14,1 ± 10,7	9,8 ± 6,1	0,020	0,92 (p = 0,012)
menor pNN50 (%)	1,44 ± 4,35	0,43 ± 1,60	0,165	
FC média (bpm)	69,7 [67,6-71,9] ^¥^	69,9 [66,6-73,3] ^¥^	0,940	
SDNN 24h (ms)	92,3 [84,6-100,7] ^¥^	78,2 [67,0-91,2] ^¥^	0,046	0,99 (p = 0,123)
SDANN 24h (ms)	80,2 [73,2-87,9] ^¥^	69,8 [57,3-85,0] ^¥^	0,200	
rMSSD 24h (ms)	26,6 [23,0-30,7] ^¥^	24,6 [18,7-32,5] ^¥^	0,620	
BVP	15 [1-181] ^¥^	156,5 [22-678] ^¥^	0,002	1,0003 (p = 0,11)
BSP	60 [22-90,5] ^¥^	94 [33-161] ^¥^	0,206	

SDNN 24h: desvio padrão de todos os intervalos RR normais gravados em 24 horas; SDANN 24h: desvio padrão das médias dos intervalos RR normais, a cada 5 minutos, em 24 horas; rMSSD 24h: raiz quadrada da média do quadrado das diferenças entre intervalos RR normais adjacentes em 24 horas; IM: infarto do miocárdio; DRC: doença renal crônica; FC: frequência cardíaca; BVP: batimentos ventriculares prematuros; BSP: batimentos supraventriculares prematuros; AVC: acidente vascular cerebral. ¥ teste de Mann-Whitney.

### Análise das variáveis com significância estatística na curva ROC

As variáveis contínuas que foram associadas com mortalidade por todas as causas foram dicotomizadas usando a curva ROC. A área sob a curva (AUC), os pontos de corte, e a significância estatística dessa análise estão resumidos na Tabela 4 . Os pontos de corte foram definidos automaticamente identificando-se o índice de Youden.

**Tabela 4:** – Identificação de pontos de corte para as variáveis contínuas

	AUC (IC 95%)	Ponto de corte	p	Sensibilidade	Especificidade
Menor SDNN (ms)	0,313 (0,213-0,413)	≤12ms	0,001	58,97%	68,83%
Menor rMSSD (ms)	0,332 (0,230-0,434)	≤12ms	0,003	87,18%	44,16%
Idade (anos)	0,658 (0,558-0,759)	>69 anos	0,006	82,05%	44,16%
FEVE (%)	0,319 (0,217-0,432)	≤57%	0,002	64,10%	71,43%

SDNN 24h: desvio padrão de todos os intervalos RR normais gravados em 24 horas; SDANN 24h: desvio padrão das médias dos intervalos RR normais, a cada 5 minutos, em 24 horas; rMSSD 24h: raiz quadrada da média do quadrado das diferenças entre intervalos RR normais adjacentes em 24 horas; FEVE: fração de ejeção do ventrículo esquerdo; AUC: área sob a curva.

### Regressão de Cox com variáveis estatisticamente significativas

Um modelo de regressão de Cox foi construído com a variável insuficiência renal crônica e as variáveis dicotomizadas de acordo com o valor recomendado pela curva ROC. O modelo está apresentado na Tabela 5 .

**Tabela 5:** – Regressão de Cox com as variáveis que apresentaram significância estatística no modelo univariado

	b	HR (IC 95%)	p
Menor SDNN ≤ 12 ms	-0,03929	0,91 (0,47-1,94)	0,9135
Menor rMSSD ≤ 12 ms	1,7126	5,54 (2,04-15,08)	0,0008
DRC	0,1643	1,18 (0,45-3,06)	0,7372
Idade > 69 anos	1,3747	3,95 (1,64-9,52)	0,0023
FEVE ≤ 57%	1,5472	4,70 (2,38-9,28)	<0,0001

SDNN: desvio padrão de todos os intervalos RR normais gravados em 24 horas; rMSSD: raiz quadrada da média do quadrado das diferenças entre intervalos RR normais adjacentes em 24 horas; DRC: doença renal crônica; FEVE: fração de ejeção do ventrículo esquerdo.

### Construção do modelo preditivo

Os parâmetros que foram avaliados usando regressão de Cox e que mostraram significância estatística foram considerados no modelo preditivo. Para a adição do ponto de corte, cada variável recebeu o valor inteiro correspondente ao seu coeficiente beta obtido na regressão de Cox ( Tabela 5 ). Assim, o modelo preditivo foi construído ( Tabela 6 ).

**Tabela 6:** – Modelo preditivo de morte

Menor rMSSD ≤ 12ms?	1
FEVE ≤ 57%?	1
Idade > 69?	1
ESCORE	SOMA

rMSSD: raiz quadrada da média do quadrado das diferenças entre intervalos RR normais adjacentes em 24 horas

**Figura 2:**
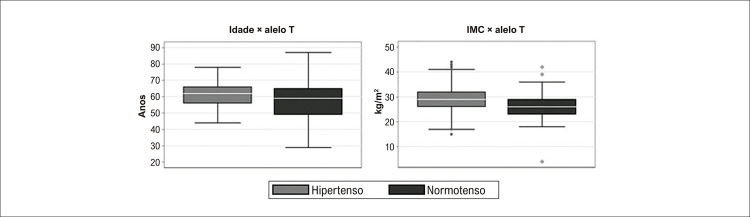
– Curvas de sobrevida de acordo com o modelo preditivo construído.

Esse escore foi avaliado usando a curva ROC para estimar sua acurácia e determinar o melhor ponto de corte. Um escore <3 mostrou uma AUC = 0,802 (IC95% 0,72-0,87) para mortalidade por todas as causas, com uma sensibilidade de 46,15% e especificidade de 97,4%. Esse escore também foi avaliado usando a regressão de Cox após a dicotomização proposta pela curva ROC. Os pacientes com um escore de três, comparativamente àqueles com um escore <3 ( Figura 2 ), tinham uma probabilidade 9,3 maior de evoluírem a óbito (HR 9,31; IC95% 4,89-17,75).

## Discussão

Morte súbita cardíaca ocorre frequentemente em pacientes com IC. ^7 , 8^ Estudos anteriores indicaram que a VFC pode predizer morte súbita cardíaca e morte por todas as causas. ^9 - 17^ Apesar disso, a análise da VFC não foi incorporada à prática clínica atual, nem como um modelo prognóstico nem como uma estratégia terapêutica que poderia reverter esse cenário.

As duas principais causas de morte súbita em pacientes com IC são IC terminal e eventos arrítmicos. ^18^ Em ambas as situações, o sistema autônomo está comprometido. Assim, a avaliação da VFC por Holter 24horas agrega informação sobre o status autonômico, permitindo a identificação de pacientes com risco aumentado.

Nosso estudo analisou os casos suspeitos e confirmados de IC em pacientes internados com IC descompensada ou síncope, e que se submeteram ao exame de Holter 24 horas. Essa abordagem metodológica permitiu a inclusão dos pacientes sem IC, e assim, a formação de um grupo controle com características similares às da população alvo (pacientes com IC).

A maioria dos pacientes apesentou função ventricular preservada (63,8%), e 64,8% desses pacientes não apresentaram IC. Apesar disso, havia indivíduos com alterações na VFC nesse subgrupo. Usando a subdivisão clássica proposta por Nolan et al., ^19^ somente 41,6% dos pacientes sem IC apresentavam SDNN 24h> 100 ms na presente amostra. Esse achado pode ser explicado pela idade avançada dos pacientes.

Analisando os parâmetros de longo prazo para o desfecho primário, somente SDNN de 24 horas e o número de BVP mostraram diferença estatisticamente significativa. No entanto, após a análise de regressão univariada de Cox, nenhum desses foi selecionado para o modelo multivariado. A literatura diverge quanto aos parâmetros de longa duração na predição de morte por todas as causas e morte cardiovascular. Sandercock e Brodie ^20^ publicaram uma revisão sistemática de estudos que analisaram o papel da VFC em diferentes tipos de morte em pacientes com IC. Vários estudos indicaram que parâmetros de longo prazo são preditores de morte cardiovascular e por todas as causas nessa população. No entanto, não só as metodologias, como os parâmetros usados e os pontos de corte eram diferentes. Usando os pontos de corte propostos por Nolan et al., ^19^ no presente estudo, uma proporção maior de óbitos também foi observada na população com SDNN 24h < 50 ms (66,7%) em comparação aos pacientes com SDNN entre 50 e 100 ms (36,2%) e SDNN > 100 ms (18,6%), com p = 0,0027. Na análise da curva ROC, uma AUC de 0,621 foi obtida com o ponto de corte proposto ≤ 98 ms, com 77,1% sensibilidade e 46,7% de especificidade. Utilizando o corte proposto por Nolan na regressão univariada de Cox, a população com SDNN < 50 ms apresentou maior risco de morte (HR 4,81 [IC95% 1,90-12,2]). Contudo, a inclusão dessa informação no modelo preditivo não aumentou a capacidade preditiva do escore (AUC: 0,809 x 0,802, p = 0,45).

Quatro variáveis foram analisadas usando a curva ROC para criar o modelo preditivo de morte por todas as causas: menor SDNN em 10 minutos, menor rMSDD em 10 minutos, idade, e FEVE. Essas variáveis foram incluídas no modelo de regressão multivariada de Cox como variáveis dicotômicas. Após a análise, somente o rMSSD em 10 minutos mais baixo, idade, e FEVE foram preditores independentes de mortalidade por todas as causas. O escore construído com essas variáveis mostraram uma boa capacidade preditiva, com uma área sob a curva ROC de 0,802. Ouwerkerk et al. ^21^ compararam modelos preditivos de morte ou hospitalização por IC em uma revisão sistemática que encontrou 117 modelos diferentes em 55 artigos. Quanto ao desfecho mortalidade, os autores relataram uma AUC média de 0,71 ± 0,001, mostrando que os modelos disponíveis para predizer mortalidade até o momento da publicação têm uma acurácia moderada. O presente estudo mostrou uma acurácia superior usando somente três variáveis. Idade superior a 69 anos, FEVE ≤ 57%, e um rMSSD em 10 minutos ≤ 12ms foram associados a um risco 9,31 vezes maior nos pacientes com IC. A inclusão da rMSSD no modelo proposto foi crítica, uma vez que ela denota um tônus parassimpático. Assim, quando reduzida, mesmo que brevemente, a rMSSD mostrou uma associação direta com mortalidade por todas as causas.

Em uma revisão sistemática publicada em 2014, Wu et al. ^22^ analisaram 138 publicações sobre o uso da VFC na predição de morte súbita. Há considerável heterogeneidade no uso das variáveis da VFC, as quais foram estudadas de maneira contínua ou dicotomizada, com diferentes pontos de corte. A variável mais estudada que mostrou a maior correlação com morte súbita foi o SDNN de 24 horas. Em vários estudos, não foram encontradas correlações entre as variáveis da VFC e morte súbita; quando encontrada, foi uma correlação fraca, com um papel preditivo pequeno nos pacientes com IC. ^22^ Somente um estudo mostrou uma fraca correlação entre rMSSD e morte súbita. Nos estudos de análise multivariada, nenhum valor preditivo foi associado com essa variável. ^23^ Na nossa revisão da literatura, nenhum estudo analisou o momento de menor VFC no monitoramento de 24 horas, o que dá importância ao presente estudo.

As principais limitações deste estudo estão no seu delineamento retrospectivo, em que os pacientes se submeteram ao exame de Holter por outro motivo, gerando possível viés de seleção. A maioria das diretrizes de IC não recomendam o uso de Holter 24 horas em nenhuma situação específica. Assim, as principais indicações para o exame de Holter estão relacionadas à investigação de eventos arrítmicos, ou por sintomas característicos (palpitações ou síncope) ou por arritmias documentadas. Em ambos os casos, os pacientes têm maior risco potencial de morte súbita e distúrbios na VFC. A exclusão do Holter 24 horas com uma alta carga arrítmica da análise também pode representar uma limitação, uma vez que os pacientes com doenças potencialmente mais graves podem ter sido excluídos.

Outra limitação foi a heterogeneidade da população. Foram incluídos pacientes com diferentes causas de IC e que se submeteram ao Holter em diferentes momentos. Nem todos os pacientes apresentavam IC descompensada e, embora todos os pacientes incluídos no estudo tiveram um exame de Holter analisado, nem todos os pacientes admitidos ao hospital foram submetidos ao monitoramento na UTI ou nos 30 dias subsequentes.

Apesar do pequeno tamanho amostral, a maioria dos estudos nesta área usou um número similar ou menor que o do presente estudo. No entanto, foi possível mostrar o impacto prognóstico das mudanças encontradas na VFC nos pacientes com IC, usando um procedimento direto de avaliação alternativa. Mais estudos são necessários para confirmar esses achados.

## Conclusão

Em pacientes com suspeita ou diagnóstico confirmado de IC, admitidos no hospital por IC descompensada ou síncope, o período de menor VFC no monitoramento eletrocardiográfico, combinado com a fração de ejeção e idade foram preditores independentes de morte por todas as causas. Essas variáveis compõem um modelo preditivo de morte por todas as causas com boa acurácia.
