# Supplementary material for: Modelo Preditivo de Mortalidade por Todas as Causas em Pacientes com Insuficiência Cardíaca Usando a Variabilidade da Frequência Cardíaca
Source: Arq Bras Cardiol. 2023 Dec 7;120(11):e20220379. [Article in Portuguese] doi: 10.36660/abc.20220379 (PMC10763696; doi:10.36660/abc.20220379)
Supplement: Translation

# Translation: Predictive Model of All-Cause Death in Patients with Heart Failure using Heart Rate Variability

**Authors:** Bruno Ferraz de Oliveira Gomes, Paulo Roberto Benchimol-Barbosa, Jurandir Nadal

**Affiliations:** 1 Brazil Hospital Barra D’Or, Rio de Janeiro, RJ – Brazil; 2 Brazil Universidade Federal do Rio de Janeiro, Rio de Janeiro, RJ – Brazil; 3 Brazil Universidade do Estado do Rio de Janeiro, Hospital Universitário Pedro Ernesto – Coordenação de Medicina Clínica, Rio de Janeiro, RJ – Brazil; 4 Brazil Universidade Federal do Rio de Janeiro Instituto Alberto Luiz Coimbra de Pós-graduação e Pesquisa de Engenharia – Programa de Engenharia Biomédica, Rio de Janeiro, RJ – Brazil

**Keywords:** Heart Rate, Heart Failure, Mortality

## Abstract

**Background:**

Short and long-duration heart rate variability (HRV) data from Holter monitoring could identify predictors of all-cause death in heart failure (HF) patients.

**Objectives:**

To build a predictive model of all-cause death in patients with HF using HRV.

**Methods:**

Retrospective study including patients with suspected or confirmed HF who were admitted for decompensated HF or syncope that underwent Holter monitoring. In analysis of augmented sympathetic tonus, we evaluated the lowest HRV in nonoverlapping 10-minutes periods throughout 24h continuous electrocardiographic signal recording (short HRV variables). Variables with p<0.01 were included in a multivariate Cox regression model to determine the occurrence of the all-cause death. Variables with statistical significance in Cox regression were chosen to build the predictive model. P<0.05 was considered significant.

**Results:**

A total of 116 patients were included, mean age of 71.9±16.3 years, 45.7% men, mean follow-up of 2.83±1.27 years. Thirty-nine deaths occurred (33.6%). By comparing survivors vs. non-survivors, the variables that showed statistical significance were lowest SDNN, lowest rMSSD, age and left ventricular ejection fraction (LVEF). In Cox regression, independent predictors of all-cause death were: age>69 years (HR 3.95, 95%CI 1.64-9.52); LVEF≤57% (HR 4.70, 95%CI 2.38-9.28) and lowest rMSSD≤12ms (HR 5.54, 95%CI 2.04-15.08). An integer value was assigned to each variable. Score<3 showed AUC=0.802 (95%CI 0.72-0.87).

**Conclusion:**

In HF patients hospitalized for decompensated HF or syncope, independent long-term predictors of all-cause death were age, LVEF, and 10-minutes rMSSD. These findings indicate that even brief moments of high sympathetic tone can impact survival, specifically in the elderly and patients with HF with reduced ejection fraction.

## Introduction

The autonomic nervous system (ANS) is responsible for maintaining body homeostasis. A variety of diseases, including heart failure (HF), can cause disorders in the ANS, compromising homeostasis, and generating changes in cardiovascular physiology. ^1^

Experimental studies have shown that an improvement in the parasympathetic influence on the heart has an antiarrhythmic and antifibrillatory effect, ^2 , 3^ while sympathetic activity is generally pro-arrhythmic. ^3^ Thus, the presence of a sympathetic dominance associated with another pro-arrhythmic process (myocardial infarction or HF) can increase the risk of sudden death. ^4^

Despite the large number of tests capable of evaluating the subject’s autonomic function, there is little data showing which tests or combinations are most appropriate in different clinical situations.

Heart rate variability (HRV) is defined as the variation in the RR interval in sinus rhythm and reflects autonomic status in regulating heart rate (HR) according to its demand. Previous studies have linked a low HRV to mortality in patients after acute myocardial infarction, in HF, diabetic neuropathy, and post-heart transplantation. ^5^

When analyzed using the 24h Holter monitoring, HRV variables express HR behavior over a long period. Long-term 24-hour measurements do not detect brief situations of increased sympathetic tone. Thus, the study of short-term variables during the moment of greatest sympathetic tone (lowest HRV) can add predictive capacity for death in these patients. This study aims to identify predictors of all-cause mortality in HF patients using clinical and short- and long-term HRV data from 24 h Holter monitoring aiming to create a predictive model of all-cause death in these patients.

## Study design

### Population

Retrospective cohort study with patients admitted for decompensated HF or syncope at two private hospitals with suspected or confirmed diagnosis of HF. The selection of patients with syncope was necessary since these patients are routinely submitted to 24h Holter monitoring. Despite having a different pathophysiological mechanism, syncope can be a marker of arrhythmic events, and be involved in an increased risk of death. ^6^ The study period was from January 2014 to December 2016. Patients who underwent Holter during hospitalization or after hospital discharge (up to 30 days) in a database with 4179 exams were identified. Patients with terminal HF, evidence of another disease whose prognosis does not provide the prospect of one-year survival, acute coronary syndrome for less than two months, Holter record with recording less than 18 hours, non-sinus HR, pacemaker carriers, and total premature beats (atrial + ventricular) ≥ 5,000 were excluded.

Clinical, laboratory, and echocardiographic data of eligible patients were assessed through electronic medical records, admission form, and discharge summary. After analysis, the patient was classified as a patient without HF (without symptoms compatible with HF or echocardiogram without systolic and diastolic dysfunction or serum B-type natriuretic peptide (BNP) levels below the laboratory reference or a patient with HF.

### Signal analysis

The signals were acquired through the three-channel Holter device, model DR200/HE (NorthEast Monitoring, MA, USA). The recording bandwidth ranges from 0.05 to 70 Hz with 12-bit resolution, with a sampling rate of 180 samples per second. The signal processing was performed using a program based on the Matlab® application (The MathWorks, MA, USA). HRV indicators were recorded by two observers, blinded to the outcomes in two situations: traditional long-term parameters and short-term parameters. In the long-term parameters, the following variables were evaluated: SDNN 24h, SDANN 24h, rMSSD 24h, average HR, total premature ventricular beats (PVB), and total premature supraventricular beats (PSB). 24h Holter recording was segmented into nonoverlapping 10-minutes windows, and the window presenting with the lowest HRV was identified to assess the short duration parameters (moment of highest sympathetic tonus). Each 10-minute window was individually examined and included in the analysis if extrasystoles, artifacts, and inadequate normal beats combined corresponded to less than 5% of the window duration. The short duration variables that were generated were: the lowest SDNN 10min (lowest SDNN recorded in a 10 min window during the 24h signal); lowest rMSSD 10min (lowest rMSSD recorded in a 10 min window during the 24h signal), and lowest pNN50 10min (smallest pNN50 recorded in a 10 min window during the 24h signal).

### Clinical variables

Age, gender, cause of HF, comorbidities, and use of previous medications were evaluated. Laboratory data and echocardiographic reports were also evaluated; the first echocardiogram performed at admission was considered in the analysis.

### Outcomes

The primary outcome of this study was all-cause mortality. The minimum follow-up period in the study was 12 months.

### Ethical aspects

The study protocol was registered on Plataforma Brasil with CAAE 63827617.5.0000.5249 after approval by the local ethics committee of Hospital Copa D’Or on 04/27/2017. As this was a retrospective observational study, the informed consent term was waived by the ethics committee.

### Statistical analysis

The results were expressed as mean ± standard deviation (normal distribution) or median and interquartile range (non-normal distribution) for continuous variables and number of occurrences (with percentage) for categorical variables. Clinical, laboratory, echocardiographic characteristics, and HRV variables in survivors and deaths were evaluated. The chi-square test for categorical variables and the unpaired Student’s t-test for continuous variables were used. When the sample distribution was not normal according to Shapiro-Wilk test, the Mann-Whitney test was used. All variables with p<0.05 were assessed using a univariate Cox regression model. Variables with p<0.01 in univariate model were included in a multivariate Cox regression model to determine the occurrence of the all-cause death. HRV variables with p<0.05 in multivariate Cox’s model for the mortality outcome were also evaluated through the ROC curve to determine the optimal cut-off point, aiming to create a predictive model score. For the addition of the cut-off point, each variable was assigned the integer value corresponding to its beta coefficient obtained in the Cox regression. Survival curves were presented in the subgroups according to the predictive model.

## Results

### Flow of inclusion of patients in the study

The inclusion flow is summarized in Figure 1 . There were 2049 hospitalizations in both hospitals; of these, 206 performed Holter (10.1%). Ninety patients were excluded from the sample, totaling 116 patients for clinical evaluation. After this evaluation, 48 (41.4%) patients with compensated cardiac function during the hospitalization period and 68 (58.6%) with HF were identified. Sixty-two patients (53.4%) were admitted due to syncope.

**Figure 1:** – The flow of inclusion of patients in the study. ACS: acute coronary syndrome.

### Population characteristics

Population characteristics are summarized in Table 1 . There was a predominance of elderly and female gender. The most prevalent comorbidity was arterial hypertension, followed by diabetes and ischemic heart disease.

**Table 1:** – Clinical characteristics

	All (n=116)	No HF (n=48)	HF (n=68)	p-value*
Age (Years)	71.9 ± 16.3	68.3 ± 21.3	74.5 ± 11.0	0.042
Male gender	53 (45.7%)	15 (31.3%)	38 (55.9%)	0.007
Arterial Hypertension	90 (77.6%)	31 (64.6%)	59 (89.4)	0.001
Diabetes	39 (33.6%)	9 (18.8%)	30 (44.1%)	0.004
Ischemic Heart Disease	21 (18.1%)	0 (0.0%)	21 (31.8%)	<0.001
Chronic Renal Failure	13 (11.2%)	1 (7.7%)	12 (17.6%)	0.007
Stroke	10 (8.6%)	2 (4.2%)	8 (11.8%)	0.135
LVEF (%)	56.3±17.2	68.5±6.8	47.7±17.2	<0.001

HF: heart failure, LVEF: left ventricular ejection fraction. * comparison between HF and No HF.

Regarding ventricular function, most patients had preserved left ventricular systolic function. The use of beta-blockers was only identified in one third of the population. Few patients manifested nonsustained ventricular tachycardia (NSVT) on Holter monitoring.

Mean Holter duration was 23.23 ± 1.48 hours, and the mean follow-up was 2.83 ± 1.27 years. Most Holter monitoring studies were performed in hospital and half were performed in the intensive care unit. There were 39 deaths.

The characteristics were also assessed according to the final classification of the patients. There was no age difference between the groups. A higher prevalence of HF, arterial hypertension, diabetes, ischemic heart disease, and chronic renal failure was observed among male than female patients.

### Heart rate variability between groups

Heart failure patients had reduced HRV parameters when compared to patients without HF. There was no difference in the mean HR between these two groups. The number of PVB was higher in patients with HF ( Table 2 ).

**Table 2:** – Heart rate variability between groups

	No HF (n=48)	HF (n=68)	Valor p
SDNN 24h (ms)	88 (71-128)*	83.5 (59-109)*	0.061
SDANN 24h (ms)	79.5 (64-112)*	71.5 (49.5-91)*	0.037
rMSSD 24h (ms)	27 (15.25-43.25)*	24.5 (18-35.5)*	0.723
Lowest SDNN (ms)	16.5 (11.5-24.5)*	13.0 (8.5-16)*	0.002
Lowest rMSSD (ms)	10 (8-19.5)*	9 (7-13.5)*	0.070
Lowest pNN50 (%)	0.01 (0.001-1.17)*	0.01 (0.001-0.17)*	0.040
Mean 24h HR (bpm)	72 (64-76.5)*	68 (62-76)*	0.159
PVB	8 (0-74.8)*	152 (7-553)*	<0.001
PSB	48 (3.75-201)*	70.5 (12-318)*	0.347

HF: heart failure; HR: heart rate; PVB: premature ventricular beats; PSB: premature supraventricular beats * median and interquartile range; SDNN: Standard deviation of the NN (R-R) intervals; SDANN: Standard Deviation of the 5 minute Average NN intervals; rMSSD: Root mean square of the successive differences; pNN50: The proportion of NN50 divided by the total number of NN (R-R) intervals; NN50: The number of pairs of successive NN (R-R) intervals that differ by more than 50 ms.

### Variables that impacted death

Results related to the all-cause mortality outcome are described in Table 3 . Mean age was higher in the group of patients who died, as well as the prevalence of chronic renal failure and reduced left ventricular ejection fraction (LVEF), and these factors were statistically significant in the univariate regression of Cox. Regarding HRV parameters, those with statistical significance in the univariate Cox regression were lowest SDNN and lowest rMSSD (p<0.05 for both).

**Table 3:** – Univariate analysis for the outcome death from all causes

	Survival (n = 77)	Deaths (n = 39)	p	Cox regression Exp(b); (p)
Mean age (years)	68.6 ± 18.0	78.4 ± 9.6	0.002	1.04 (p = 0.003)
Male gender	32 (41.6%)	21 (53.8%)	0.145	
Hypertension	58 (75.3%)	32 (86.5%)	0.130	
Diabetes	24 (31.2%)	15 (38.5%)	0.280	
Previous MI	14 (18.2%)	7 (18.9%)	0.557	
CKD	3 (3.9%)	10 (25.6%)	0.001	4.5 (p<0.001)
Previous stroke	4 (5.2%)	6 (15.4%)	0.070	
Betablocker use	24 (31.2%)	14 (35.9%)	0.378	
LVEF (%)	59.9 ± 15.9	49.3 ± 17.7	0.002	0.98 (p = 0.003)
Lowest SDNN (ms)	18.7 ± 11.4	12.3 ± 5.2	0.001	0.90 (p = 0.001)
Lowest rMSSD (ms)	14.1 ± 10.7	9.8 ± 6.1	0.020	0.92 (p = 0.012)
Lowest pNN50 (%)	1.44 ± 4.35	0.43 ± 1.60	0.165	
Mean HR (bpm)	69.7 [67.6-71.9] ^¥^	69.9 [66.6-73.3] ^¥^	0.940	
SDNN 24h (ms)	92.3 [84.6-100.7] ^¥^	78.2 [67.0-91.2] ^¥^	0.046	0.99 (0.123)
SDANN 24h (ms)	80.2 [73.2-87.9] ^¥^	69.8 [57.3-85.0] ^¥^	0.200	
rMSSD 24h (ms)	26.6 [23.0-30.7] ^¥^	24.6 [18.7-32.5] ^¥^	0.620	
PVB	15 [1-181] ^¥^	156.5 [22-678] ^¥^	0.002	1.0003 (0.11)
PSB	60 [22-90.5] ^¥^	94 [33-161] ^¥^	0.206	

MI: myocardial infarction; CKD: chronic kidney disease; HR: heart rate; PVB: premature ventricular beats; PSB: premature supraventricular beats. SDNN: Standard deviation of the NN (R-R) intervals; SDANN: Standard Deviation of the 5 minute Average NN intervals; rMSSD: Root mean square of the successive differences; pNN50: The proportion of NN50 divided by the total number of NN (R-R) intervals; NN50: The number of pairs of successive NN (R-R) intervals that differ by more than 50 ms; LVEF: left ventricular ejection fraction; ¥ Mann-Whitney test.

### Analysis of statistically significant variables in the ROC curve

Continuous variables that were associated with all-cause death were dichotomized using the ROC curve. The area under the curve (AUC), the cut-off points, and the statistical significance of this analysis are summarized in Table 4 . The cut-off points were defined automatically by identifying the Youden index.

**Table 4:** – Identification of cut-off points for continuous variables

	AUC (95% CI)	Cutoff point	p	Sensibility	Specificity
Lowest SDNN (ms)	0.313 (0.213-0.413)	≤12ms	0.001	58.97%	68.83%
Lowest rMSSD (ms)	0.332 (0.230-0.434)	≤12ms	0.003	87.18%	44.16%
Age (years)	0.658 (0.558-0.759)	>69 anos	0.006	82.05%	44.16%
LVEF (%)	0.319 (0.217-0.432)	≤57%	0.002	64.10%	71.43%

LVEF: left ventricular ejection fraction; AUC: area under the curve; SDNN: Standard deviation of the NN (R-R) intervals; rMSSD: Root mean square of the successive differences.

### Cox regression with the statistically significant variables

A Cox regression model was built with the variable chronic renal failure and the dichotomized variables according to the value recommended by the ROC curve. The model is shown in the Table 5 .

**Table 5:** – Cox regression with the variables that demonstrated statistical significance in the univariate model

	b	HR (95%CI)	p
Lowest SDNN ≤ 12 ms	-0.03929	0.91 (0.47-1.94)	0.9135
Lowest rMSSD ≤ 12 ms	1.7126	5.54 (2.04-15.08)	0.0008
CKD	0.1643	1.18 (0.45-3.06)	0.7372
Age > 69 years	1.3747	3.95 (1.64-9.52)	0.0023
LVEF ≤ 57%	1.5472	4.70 (2.38-9.28)	<0.0001

CKD: chronic kidney disease; LVEF: left ventricular ejection fraction; SDNN: Standard deviation of the NN (R-R) intervals; rMSSD: Root mean square of the successive differences;

### Predictive model construction

The parameters evaluated using Cox regression and that showed statistical significance were considered in the predictive model. For the addition of the cut-off point, each variable received the integer value corresponding to its beta coefficient obtained in the Cox regression ( Table 5 ). Thus, the predictive model was built ( Table 6 ).

**Table 6:** – Predictive model of death

Lowest rMSSD ≤ 12ms?	1
LVEF ≤ 57%?	1
Age > 69?	1
SCORE	SUM

rMSSD: Root mean square of the successive differences; LVEF: left ventricular ejection fraction

This score was evaluated using the ROC curve to estimate its accuracy and determine the best cut-off point. A score <3 showed an AUC = 0.802 (95% CI 0.72-0.87) for all-cause death with a sensitivity of 46.2% and specificity of 97.4%. This score was also assessed using Cox regression after dichotomization proposed by the ROC curve. Patients with a score of three, as compared to those with a score <3 ( Figure 2 ), were 9.3 times more likely to die (HR 9.31; 95% CI 4.89-17.75).

**Figure 2:** – Survival curves according to the built predictive model.

## Discussion

Sudden cardiac death frequently occurs in HF patients. ^7 , 8^ Previous studies have indicated that HRV can predict sudden cardiac death, as well as all-cause death. ^9 - 17^ Despite this, HRV analysis has not been incorporated into current clinical practice, either as a prognostic model or a therapeutic strategy that could reverse this scenario.

The two leading causes of sudden death in patients with HF are terminal HF and arrhythmic events. ^18^ In both situations, the autonomic system is compromised. Thus, the assessment of HRV through the 24-hour Holter aggregates information on autonomic status, allowing the identification of patients at increased risk.

Our study analyzed suspected and confirmed HF in hospitalized patients with decompensated HF or syncope, who underwent 24-hour Holter monitoring. This methodological approach allowed the inclusion of patients without HF, and hence the formation of a control group with similar characteristics to the target population (HF patients).

Most patients had preserved ventricular function (63.8%), and 64.8% of these patients did not have HF. Despite this, there were individuals with changes in HRV in this subgroup. Using the classic subdivision proposed by Nolan et al., ^19^ only 41.6% of patients without HF had SDNN 24h> 100 ms in the present sample. This finding can be justified by the patients’ advanced age.

Analyzing the long-term parameters for the primary outcome, only the SDNN 24h and the number of PVB showed statistical difference. However, after univariate Cox regression analysis, none of them were selected for the multivariate model. The literature differs regarding the long-term parameters in predicting all-cause death and cardiovascular death. Sandercock and Brodie ^20^ published a systematic review of studies that analyzed the role of HRV in different types of death in patients with HF. Several studies have indicated that long-term parameters predict cardiovascular and all-cause death in this population. However, not only the methodologies, but also the parameters used, and their cut-off points were different. Using the cut-off points proposed by Nolan et al. ^19^ in the present study, a higher proportion of deaths was also observed in the population with SDNN 24h < 50 ms (66.7%) compared with SDNN between 50 and 100 ms (36.2%) and SDNN > 100 ms (18.6%) with p = 0.0027. In the ROC curve analysis, an AUC of 0.621 was obtained, with the proposed cut-off point of ≤ 98 ms, with 77.1% sensitivity and 46.7% specificity. Using the cut-off proposed by Nolan in Cox univariate regression, the population with SDNN <50 ms had a higher risk of death (HR 4.81 [95% CI 1.90-12.2]). However, the inclusion of this information in the predictive model did not increase the predictive capacity of the score (AUC: 0.809 x 0.802, p = 0.45).

Four variables were analyzed using the ROC curve to create the predictive model of all-cause death: lowest SDNN 10min, lowest rMSSD 10min, age, and LVEF. These variables were included in Cox multivariate regression model as dichotomized variables. After the analysis, only the lowest rMSSD 10min, age, and LVEF were independent predictors of all-cause mortality. The score constructed with these variables showed a good predictive capacity, with an area under the ROC curve of 0.802. Ouwerkerk et al. ^21^ compared predictive models of death or hospitalization for HF in a systematic review that found 117 different models in 55 articles. As for the mortality outcome, the authors reported a mean AUC of 0.71 ± 0.001 showing that the models available to predict mortality to the time of publication have only moderate accuracy. The present study showed superior accuracy using only three variables. Age greater than 69 years, LVEF ≤ 57%, and a lowest rMSSD 10min ≤ 12ms were associated with a 9.31 time-greater risk of death in patients with HF. The inclusion of rMSSD in the proposed model is critical since it denotes a parasympathetic tone. Thus, when reduced even briefly, rMSSD was directly associated with all-cause death.

In a systematic review published in 2014, Wu et al. ^22^ analyzed 138 publications on the use of HRV in the prediction of sudden cardiac death. There is considerable heterogeneity regarding the use of HRV variables, which have been studied continuously or dichotomized, with different cut-off points. The most studied variable, which showed the highest correlation with sudden cardiac death, was the 24h SDNN. In several studies, no correlations were found between HRV variables and sudden cardiac death; when found, this correlation is weak and has a minor predictive role in patients with HF. ^22^ Only one study showed a weak correlation between rMSSD and sudden cardiac death. In the multivariate analysis studies, no predictive value was associated with this variable. ^23^ In our literature review, no study analyzed the moment of lowest HRV in 24-hour electrocardiographic monitoring, which gives relevance to the present study.

The main limitations of this study lie in the fact that it has a retrospective design in which patients underwent Holter for another purpose, generating possible selection bias. Most HF guidelines do not recommend the use of 24h Holter in any specific situation. Thus, the main indications for Holter monitoring are related to the investigation of arrhythmic events, either by characteristic symptoms (palpitations or syncope) or by documented arrhythmias. In both cases, patients are potentially at greater risk of sudden death and disturbances in HRV. The exclusion of 24h ECG Holter with a high arrhythmic burden from the analysis may also represent a limitation, as patients with potentially more severe diseases may have been excluded.

Another limitation was the heterogeneity of the population. Patients with different causes of HF and who underwent Holter at different moments were included. Not all had decompensated HF and, although all patients included in the study had a Holter monitoring analyzed, not all patients admitted to the hospital underwent monitoring in the ICU or for 30 days afterward.

Despite the small sample size, most studies in this area used a number similar to or less than the present study. Nonetheless, it was possible to show the prognostic impact of changes found in HRV in patients with HF, using a straightforward alternative assessment procedure. Further studies are needed to confirm these findings.

## Conclusion

In patients with suspected or confirmed HF who were admitted for decompensated HF or syncope, the period of lowest HRV in 24-hour electrocardiographic monitoring combined with the ejection fraction and age were independent predictors of all-cause death. These variables compose a predictive model of death from all causes with good accuracy.
